# Intestinal Obstruction in Pregnancy: A Case Report

**DOI:** 10.1155/2013/564838

**Published:** 2013-02-06

**Authors:** Shakina Rauff, Stephen Kin Yong Chang, Eng Kien Tan

**Affiliations:** ^1^Department of Obstetrics & Gynaecology, National University Hospital, 5 Lower Kent Ridge Road, Singapore 119074; ^2^Division of Hepatobiliary and Pancreatic Surgery, Liver Transplant Programme, Department of Surgery, National University Hospital, Singapore 119074

## Abstract

*Background*. Intestinal obstruction in pregnancy is uncommon. The condition is associated with significant maternal and fetal mortality. The delay in diagnosis is due to nonspecific symptoms and a disinclination to carry out radiologic investigations in pregnancy. *Case*. A 39-year-old lady at 32 weeks of gestation presented with abdominal pain and nausea. Her symptoms worsened during admission. A computed tomography (CT) scan showed dilated small bowel loops suggestive of intestinal obstruction. She eventually underwent a laparotomy as conservative measures failed. *Conclusion*. A high index of clinical suspicion is required to diagnose intestinal obstruction in pregnancy. Prompt diagnosis should be made and the appropriate treatment instituted. Surgical intervention should be performed if necessary as further delay only results in increased morbidity and mortality.

## 1. Case Report

A 39-year-old patient, para 1, presented at 32 weeks' gestation with colicky abdominal pain and nausea. She had no fever, vomiting, constipation, or urinary symptoms. Her pregnancy had been uneventful and she had no prior medical or surgical history.

On admission, she was afebrile with normal pulse and blood pressure. The uterus was soft and there were no palpable contractions. She had epigastric tenderness, but normal bowel sounds. CTG was reassuring with irregular uterine activity but the cervix was closed. Full blood count, liver and renal panel were normal.

The working diagnosis was threatened preterm labour versus intestinal colic. She was commenced on IV fluids, antiemetics, antispasmodics, analgesia, and oral nifedipine for tocolysis. Fetal lung maturation was induced with dexamethasone. 

**Figure 1:**
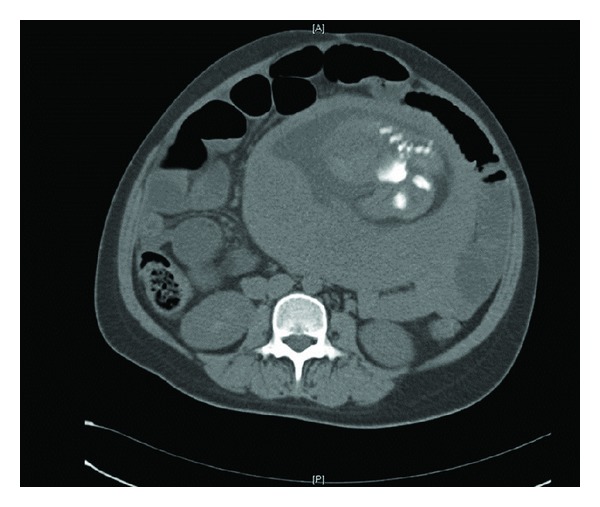
CT scan of patient showing dilated small bowel loops and part of the fetus.

The initial abdominal ultrasound was normal. Over the next 24 hours, she developed fever with increasing abdominal pain, distension and started vomiting large amounts of bilious fluid. Surgical referral suggested subacute intestinal obstruction and she was managed conservatively with nasogastric suctioning, IV fluids, and antibiotics. A computed tomography (CT) scan with contrast showed nonspecific mild dilatation of the distal jejunal loops and proximal ileum (Figure 1).

Over the next 72 hours, her symptoms worsened with high volumes of nasogastric aspirate. A subsequent laparotomy confirmed a mesenteric band constricting the distal ileum, with proximal small bowel dilatation and collapsed distal ileum. The mesenteric band was lysed and the bowel retrogradely decompressed. The abdomen was closed with loop PDS. 

She recovered well and went home a week later. At 39 weeks, she went into labour and had an uneventful vaginal delivery. She delivered a healthy baby boy weighing 3200 gm and with Apgar scores of 9 and 9 at 1 minute and 5 minutes of life, respectively. 

## 2. Discussion

Intestinal obstruction (IO) in pregnancy is rare at 1 in 2500 [1] to 1 in 16709 [2] deliveries. Although uncommon, IO in pregnancy carries significant maternal (6%) and fetal (26%) mortality [3]. Often, this is due to delay in diagnosis and treatment. Furthermore, there is a reluctance to utilise radiation-based investigations. This was the first case of IO in pregnancy recorded in our hospital in the last 10 years (2500 deliveries annually). 

### 2.1. Causes

Adhesions are the commonest cause of IO in pregnancy and account for more than half the causes found at laparotomy. The incidence and complication rates increase with gestational age, particularly in the third trimester. The risk of gestational IO increases as the uterus enlarges. Other causes include volvulus (23%), intussusception (5%), hernia (3%), carcinoma (1%), appendicitis (1%), and idiopathic “ileus” (8%) [3].

### 2.2. Diagnosis

The diagnosis of IO in pregnancy is difficult. Signs of acute abdomen may not be as prominent in the pregnant abdomen when compared to the nonpregnant one [4], due to the stretched anterior abdominal wall being less sensitive to parietal peritoneal irritation. 

Our patient presented with generalised abdominal pain and nausea which progressed to vomiting and abdominal distension, indicating an obstructive pathology. She had no previous surgical history to suspect adhesions as a cause for intestinal obstruction. In suspected intestinal obstruction, CT, rather than MRI, has recently emerged as the diagnostic modality of choice. In contrast to MRI, CT involves ionizing radiation, whereas MRI requires gadolinium contrast, with an uncertain safety profile in pregnancy. However, CT is justified in our case as the risks of radiation are outweighed by the maternal-fetal risks of missing the diagnosis.

### 2.3. Management

Management of IO in pregnancy is similar to nonpregnancy. Clinical suspicion is vital and joint management between surgeons and obstetricians is crucial. The basis of treatment is timely surgery, minimising delays in decision. 

The initial treatment consists of nasogastric aspiration with aggressive IV fluids to correct electrolyte disturbances. CT with contrast gives the most diagnostic information on the level and cause of obstruction.

Failure of conservative treatment and demonstration of complete obstruction on CT are indications for early surgery as persistence will contribute to an increase in mortality and morbidity. Perinatal death from hypoxia secondary to maternal hypovolaemia, sepsis, and peritonitis has been reported [5]. Maternal nutritional deficiencies can occur if the patient is kept nil per oris (NPO) for a protracted period. Surgery should be performed via a midline incision to allow adequate exposure and complete exploration of abdomen with minimal manipulation of uterus. The entire bowel must be examined for other areas of obstruction and viability. Segmental resection with or without anastomosis may be necessary in the presence of gangrenous bowel. In our patient, conservative measures failed after 3 days. At laparotomy, all she required was lysis of a mesenteric band at the ileocaecal junction and retrograde decompression of the bowel.

If fetal distress is present or if there is inadequate exposure at laparotomy, delivery by caesarean section should precede the relief of obstruction. There was also a potential dilemma that the primary abdominal incision closure would not be possible, therefore exposing the fetus to risks of ensuing sepsis and peritonitis, necessitating an early caesarean section. Fortunately, after retrograde decompression of the small bowel, closure was possible. 

## 3. Conclusion

IO in pregnancy is rare. A high degree of suspicion is crucial, especially in patients with previous abdominal surgery. The high morbidity and mortality rates meant that radiological investigations and surgery should not be delayed. An additional learning point from this case would be that in an obstetric patient without any surgical history who presents with abdominal pain, one should always consider rarer surgical causes other than merely obstetric or gynaecological causes of pain.
